# Supplementary figures and images for: Mixed-species flock sizes and compositions influence flock members’ success in three field experiments with novel feeders
Source: PLoS One. 2024 May 9;19(5):e0301270. doi: 10.1371/journal.pone.0301270 (PMC11081282; doi:10.1371/journal.pone.0301270)

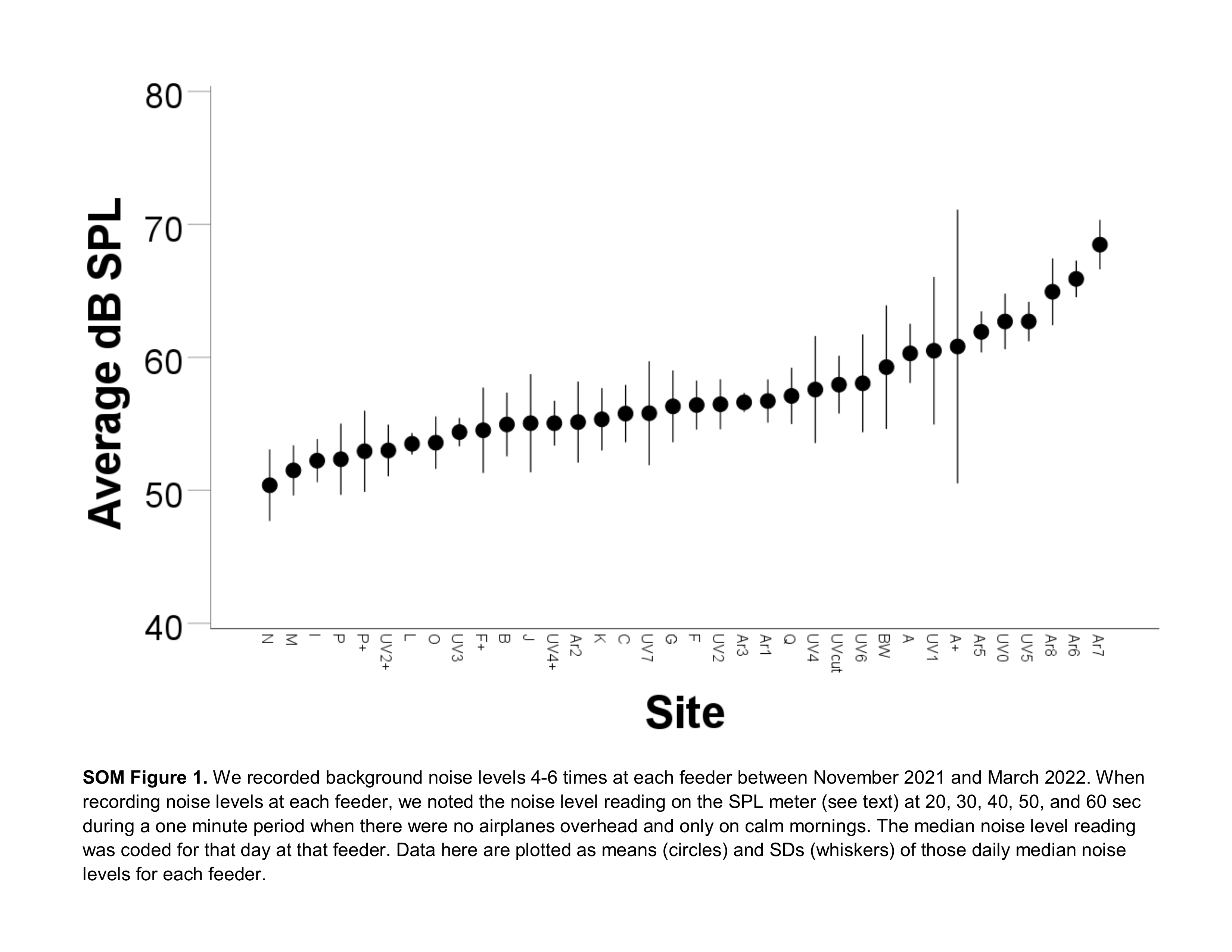

Supplement: S1 Fig — When recording noise levels at each feeder, we noted the noise level reading on the SPL meter (see text) at 20, 30, 40, 50, and 60 sec during a one minute period when there were no airplanes overhead and only on calm mornings. The median noise level reading was coded for that day at that feeder. Data here are plotted as means (circles) and SDs (whiskers) of those daily median noise levels for each feeder. (TIF) [file pone.0301270.s001.tif]
